# Family carers’ perspectives of the Alzheimer Café in Ireland

**DOI:** 10.12688/hrbopenres.13040.2

**Published:** 2020-08-17

**Authors:** Áine Teahan, Christine Fitzgerald, Eamon O'Shea

**Affiliations:** 1 Centre for Economic and Social Research on Dementia, NUI Galway, Galway, Ireland

**Keywords:** Dementia, Family carers, Psychosocial, Alzheimer Café

## Abstract

**Background:** The Alzheimer Café is a psychosocial intervention shown to have benefits for family carers of people with dementia. Family carers experience a period of change across all aspects of their lives following the dementia diagnosis, and require new skills and tools to navigate these new landscapes. The objective of this research was to investigate family carers’ perspectives of the Alzheimer Café in Ireland, and explore how attendance may translate into broader benefits in their lives. This paper also provides an overview of Alzheimer Café models, which exist internationally.

**Methods:** Semi-structured interviews were conducted with nine family carers of people with dementia who were currently attending or had attended an Alzheimer Café in the preceding six months. The research was conducted in three Alzheimer Cafés in Ireland. Data analysis was conducted using Braun and Clarke’s six step thematic analysis framework.

**Results:** Community, atmosphere, activity and information were described as core features of the Alzheimer Café in Ireland. The Alzheimer Café was described as a community with a good atmosphere encompassing emotional support, friendship, equality and inclusion. Family carer also highlighted Alzheimer Cafés could potentially facilitate wider community awareness and engagement. The Alzheimer Café was shown to provide an activity which facilitated relationship building within care dyads as well as with other attendees. Several information streams were identified, including guest speaker input, attendees’ shared experiences, and specific advice from healthcare professionals.

**Conclusion:** The Alzheimer Café offers strong personal support to family carers of people with dementia. Our findings suggest that Alzheimer Cafés can build family carers’ capacity to manage new social, environmental and cultural challenges associated with dementia. While it is important the Alzheimer Café is enjoyable, has useful information and is supportive, it is equally important that these features generate sustained improvements for family carers external to the Alzheimer Café.

## Introduction

The Alzheimer Café is a psychosocial intervention shown to have a positive impact on different aspects of family carers and people with dementias’ lives ( Dow *et al*., 2011; Greenwood *et al*., 2017; Merlo *et al*., 2018; Winterton & Warburton, 2011). The Alzheimer Café model was developed by Bére Miesen in the Netherlands in the 1970s to address the lack of psychosocial supports within the largely medicalised approach to dementia care at the time. The original Alzheimer Café model had three mains aims: to provide information about medical and psychosocial aspects of dementia; to speak openly about challenges associated with dementia; and to reduce isolation for people with dementia and their families ( Miesen & Blom, 2001).

This original model, sometimes referred to as the ‘European Alzheimer Café model’ ( Dementia Action Collaborative, 2018; Wisconsin Alzheimer's Institute, 2017), adheres to a set of strict quality control criteria. Given the wider international uptake of the Alzheimer Cafés in regions, including America, Australia and Japan, variations in the delivery of the model have, not surprisingly, emerged. One of the most notable divergences has been the development of an ‘American Alzheimer Café model’ ( Lokvig, 2016). Where the European model gives considerable attention to the provision of information and education on various aspects of dementia, the American model emphasises socialising, creative engagement and developing connections with local community cultural resources. While education is not a key component of the American Alzheimer Café model, general information about dementia is sometimes available in the form of leaflets ( McFadden & Koll, 2014).

Other countries have adopted elements of the European and American model when delivering Alzheimer Cafés. In Australia, some Alzheimer Cafés have been rebranded Memory Lane Cafés and are largely delivered as a social and educational service. The Australian Memory Lane Café model aims to promote social inclusion, prevent social isolation and improve the social and emotional well-being of the people with dementia, family carers and service providers in attendance ( Dow *et al*., 2011; Mather, 2006). Similar to the European model, the Memory Lane Café has an informational and a social focus but is more flexible in delivering these elements of the model. The Memory Lane Café also incorporates a counselling component in the form of a counsellor. The role of counsellor varies in different Memory Lane Cafés sometimes facilitating private discussion in person or over the phone, in other Memory Lane Cafés attendees are not aware that the staff member is a counsellor ( Dow *et al*., 2011). The Japanese government has recognised over 655 Dementia Cafés and has formally advocated for the establishment of these Dementia Cafés through policy documents ( Fukui *et al*., 2019). There is considerable variation in how Dementia Cafés are delivered throughout Japan, often including diverse combinations of social, entertainment and/or educational aspects ( Fukui *et al*., 2019).

Despite the international variations of models, Alzheimer Cafés have been shown to reduce social isolation, facilitate social network development and act as useful information sources ( Capus, 2005; Dow *et al*., 2011; Fukui *et al*., 2019; Greenwood *et al*., 2017). In addition, the Alzheimer Café helps attendees to ‘normalise the experiences related to dementia’ ( Capus, 2005; Greenwood *et al*., 2017). Research has shown different benefits for family carers and people with dementia; while carers benefit from information and peer support ( Akhtar *et al*., 2017), people with dementia enjoy the atmosphere and activity aspects of the Café ( Capus, 2005). One quantitative study in Italy reported improved measures of well-being, vitality, and emotional burden for family carers following engagement with the Alzheimer Café ( Merlo *et al*., 2018).

In Ireland the overall provision of services for dependent older people varies considerably and is often described as a postcode lottery ( Smith *et al.*, 2019). It is difficult to comment on the relationship between community-based service provision and Alzheimer Cafés given the fragmented provision of services in general. A recent study mapped dementia-specific service provision in Ireland, these findings indicate that healthcare regions with higher availability of dementia-specific services are more likely to provide an Alzheimer Café. However, this was not the case for all healthcare regions ( National Dementia Office *et al*., 2016). In 2011, the first Irish Alzheimer Café was established in the Dublin area. Since then, 26 Alzheimer Cafés have been set up throughout Ireland, 20 of which were still in operation in 2019. Given the significant uptake in establishing Alzheimer Cafés across Ireland, a temporary Alzheimer Café co-ordinator role has been funded through the National Dementia Office to develop a national Alzheimer Café network. However, the ad-hoc growth in Alzheimer Cafés in Ireland has resulted in considerable variation in delivery and governance across existing Alzheimer Cafés. These differences not only include variations in organisational elements such as branding, venue (type and scale), funding models and local governance, but also include differences in conceptual underpinnings and guiding principles. Consequently, creating a national governance model and national guidelines that work for all Alzheimer Cafés is a complex challenge.

As so many different approaches to Alzheimer Cafés in Ireland currently exist, it is important that the development of national guidelines is evidence-based. Understanding the experiences of family carers and people with dementia is central to ensure relevance and sustainability of the service. International research suggests people with dementia and family carers derive different benefits from psychosocial programmes ( Fukui *et al*., 2019; Kinsey *et al*., 2019), therefore capturing the perspectives of both people with dementia and family carer groups is essential. This paper focuses specifically on the experiences of family carers, but the work is part of a larger study exploring different stakeholder experiences including those of people with dementia. Family carers of people with dementia report significant personal challenges as a consequence of their caring role. Many family carers report problems such as: lack of information on available services; financial constraints; administrative problems accessing services; low social recognition among healthcare professionals; social isolation within their community ( Birtha & Holm, 2017). As a consequence of these challenges, family carers of people with dementia have reported significantly higher levels of depression, anxiety and loneliness ( Brennan *et al*., 2017; Lafferty *et al*., 2014). Given that family carers are a crucial component of any long-term care system in Ireland, supporting and maintaining their well-being is an important policy response to the growing prevalence of dementia and corresponding demand for long-term care.

In this context the potential benefits of Alzheimer Cafés for family carers are significant. Internationally, Alzheimer Cafés have been shown to facilitate informational support through signposting, guest speakers and access to healthcare professionals ( Takechi *et al*., 2019). Delivering information about dementia in a safe atmosphere enabled family carers to process information without feeling distressed or under pressure helping them to normalise dementia ( Greenwood *et al*., 2017; Takechi *et al*., 2018). In addition, research suggests that the opportunity to socialise with other family carers alleviated social isolation and led to the development of informal peer support networks ( Dow *et al*., 2011; Takechi *et al*., 2019). Previous research has shown that longer term interventions produce better outcomes for family carers ( Pinquart & Sörensen, 2006), consequently the on-going nature of the Alzheimer Café may be beneficial for family carers over time.

The aim of this research is to explore the experiences of family carers of people with dementia attending Alzheimer Cafés in Ireland. As well as capturing the immediate benefits associated with attending the Café, the research explores how these benefits translate into family carers’ wider interactions and experiences. A criticism of standard psychosocial programme evaluations is their tendency to focus solely on the immediate experience of individuals ( Innes, 2009), rather than engaging with the wider social, environmental and cultural implications beyond the programme itself. Many studies in this area are evaluated quantitatively, focusing on immediate improvements in outcomes such as depression ( Teahan *et al*., 2020), with complex constructs such as skill development, sense of community or network building rarely being considered. The potential of psychosocial programmes to create long-term wide-reaching benefits in people’s lives is, therefore, often under-acknowledged in research. That is why we take an ecological systems approach to understanding the personal and social impact of the Alzheimer Café on family carers, outlined in more detailed in the subsequent section.

## Methods

### Study design

Semi-structured interviews were conducted with family carers who were currently attending or had previously attended an Alzheimer Café in the preceding six months. A qualitative approach facilitated in-depth exploration and understanding of family carers’ experiences of attending the Alzheimer Café. The approach also explored the impact of Alzheimer Café attendance on family carers’ private and social lives. Interviews were conducted from April 2018 – January 2020. Ethics for the study was granted by the University Research Ethics Committee, National University of Ireland Galway, reference number 18-Jan-16.

### Recruitment

The research was conducted in three Alzheimer Café sites in Ireland. The selection of sites was based on convenience, as co-ordinators in each of the three chosen Alzheimer Cafés expressed an interest in the research topic and in facilitating the recruitment of family carers. For the purpose of recruitment, the principal researcher initially attended an Alzheimer Café meeting at each site and presented an overview of the research to those attending the Café. Family carers were invited to participate in the research at that initial meeting. If family carers expressed an interest in participating in the research, they were provided a copy of the information sheet and the consent form, their contact details were documented, and they were followed up later to arrange an interview. The study consisted of nine interviews with family carers of people with dementia, three from each Alzheimer Café site. All family carers who expressed interest in participating in the study were interviewed. For the purpose of this study, family carers were deemed eligible to participate if they were currently/had previously, provided unpaid, informal care to a person with dementia and had attended at least one Alzheimer Café meeting in the six months prior to the interview. Given the sometimes unpredictable nature of caring for a person with dementia, the timeframe of six months was chosen to avoid excluding family carers who had wished to attend but been unable to for whatever reason. This timeframe also avoided excluding family carers who had only recently started attending but felt their attendance had a meaningful impact at this early stage. Family carers in this study were provided with an information sheet and consent form in advance of their interview. On the day of the interview, family carers had the opportunity to ask the researcher questions in relation to either document prior to signing the consent form.

### Interview guide

The interview guide ( Table 1) was informed by relevant literature in the Alzheimer Café field ( Akhtar *et al*., 2017; Capus, 2005; Dow *et al*., 2011; Greenwood *et al*., 2017; Merlo *et al*., 2018). An ecological systems framework ( Bronfenbrenner, 1979) also influenced the approach taken to developing the interview guide. An ecological framework embeds each family carer within a wide range of interacting economic, social and cultural systems. While ecological systems frameworks have been used to explore the impact of multiple environmental influences on different populations including palliative care ( Chandran *et al*., 2016; Wittenberg-Lyles *et al*., 2010), community-based services ( Jani *et al*., 2012), and team-based psychotherapy ( Hyer *et al*., 2011), we do not develop an ecological framework specifically for our work on Alzheimer Cafés. Consultations with family carers during early design phases of the research highlighted practical challenges for interviewees in equating the Alzheimer Café with wider ecological systems thinking in the allocated interview time. Consequently, a more organic approach was taken with the interview guide where prompts were used to encourage family carers to identify wider social issues (system organisation, culture, society, public policy etc.) they had experienced while caring for a person with dementia. Through this reflection interviewees then considered the impact attending the Alzheimer Café had, or did not have, or could potentially have in these contexts. For example, although not explicitly asking about the healthcare system organisation- asking family carers how they engaged with healthcare professionals in the Alzheimer Café opened up discussions about the broader health system, specifically how family carers fared while navigating this system and if attending the Alzheimer Café had helped them acquire skills to navigate these systems. Another example included asking family carers about their social life which drew responses on wider community stigma associated with dementia and prompted discussion on the Alzheimer Café in this context.

**Table 1.** Interview guide prompt questions.

Interview Guide
1. What aspects of an average Café do you enjoy/feel are most beneficial for you?
2. Do you think attending the Café has had an impact on your own personal and social life? If so, in what ways? Prompts Meeting other family carers and people with dementia Relationship-building with person with dementia Explaining dementia to friends and family Lack of understanding and stigma in community Looking after your own well-being
3. Are there any instances where you found the Café to be particularly useful or supportive in relation to the practicalities of dementia care? Prompts Learning about dementia Regular informal contact with healthcare specialists Learning about services and supports available (health, social care, legal) Navigating these services and supports
4. Are there any services or supports that you have started to use as a result of attending the Café?
5. Have there been any negative aspects associated with your use of the Alzheimer Café?

### Data collection

Pilot interviews were conducted with two family carers and their feedback was integrated to develop the final interview guides (see Table 1). Feedback from pilot interviews resulted in changes in wording and ordering of some questions as well as the inclusion of additional prompts. Following each interview, the authors identified any novel themes, which were integrated into subsequent interviews as prompts. For example, general community awareness was mentioned in the second interview, this was included as a prompt in each of the following interviews. The interviews were semi-structured to allow the interviewer to explore topics which were particular to each interviewee, but also facilitating comparison of key questions. In-depth interviews were conducted by the primary author (ÁT), a PhD researcher with a background in Psychology and Economic research. Interviews were audio-taped and transcribed with the permission of the participants. Two participants preferred not to be audio-taped, in these cases extensive notes were taken throughout the interview. Interviews were conducted at a location of choice of the interviewee, including Alzheimer Café venues, quiet public settings and the participants’ own home. The average interview lasted 37 minutes.

### Data analysis

Data analysis was conducted using Braun and Clarke’s six step framework for thematic analysis using NVivo 10 qualitative data software ( Braun & Clarke, 2013).

The first step in the analysis involved in-depth reading and re-reading of the interview data simultaneously generating an initial list of ideas and note, this phase was ongoing throughout the data analysis process. Once the initial list of ideas and notes had emerged from reading and re-reading the data, the principal researcher re-read the transcripts alongside her own reflective notes and observations. These reflective notes and observations were compared and integrated into the initial list of ideas and notes.

The second phase involved generating codes and initial themes that encapsulated responses relevant to the research question. This phase involved coding individual transcripts and identifying emergent themes. Once themes had been identified for each individual transcript, the transcripts were analysed on a group level consisting of all transcripts, looking at relationships and links between emergent themes identified. Tentative descriptions for the emergent themes were developed at this point supported by direct quotes from interviewees.

Once all the interviews had been coded and initial themes identified, the researchers moved onto the third phase which involved compiling the codes and initial themes into a provisional overarching framework comprising main provisional themes. Using this framework, subthemes were identified and collapsed under provisional theme headings based on their content and relevance to the research question.

Steps four and five involved ongoing analysis to ensure the refinement of themes, including additional focus on the clarity of the descriptions and names of each theme. Each individual transcript was re-read in the context of the main provisional theme headings to ensure that initial codes still resonated with theme names and descriptions. This was part of an iterative process of testing and refining the coding system. The sixth step involved the using the consolidated criteria for reporting qualitative research (COREQ) ( Tong *et al*., 2007) statement to report and write up our final thematic analysis.

## Results

Nine family carers of people with dementia participated in individual semi-structured interviews. This sample comprised of spousal carers (N=5) and adult child (N=4) family carers, the majority of this sample was female (N=7). The average duration of attendance was 3.4 years (see Table 2) and the majority of family carers said they were regular attendees. Four core themes emerged from the analysis: Community, Atmosphere, Activities and Information.

**Table 2.** Duration of attendance.

	Person cared for	Duration of attendance	Frequency of attendance
Family carer 1	Husband	2 months	New attendee
Family carer 2	Mother	6 years	Regular attendee
Family carer 3	Husband	1.5 years	Regular attendee
Family carer 4	Mother	6 years	Regular attendee
Family carer 5	Husband	3 years	Regular attendee
Family carer 6	Husband	6 years	Regular attendee
Family carer 7	Husband	1 year	Regular attendee
Family carer 8	Mother	6 years	Regular attendee
Family carer 9	Wife	1 year	Regular attendee

### Community

The Alzheimer Café provided family carers with an opportunity to be part of a new supportive community. This community network consisted of people with dementia, other family carers, staff and volunteers. The sense of community fostered by the Alzheimer Café was supported in several ways through different activities and connections.

Emotional support networks were an important component of the Alzheimer Café, particularly in alleviating social isolation for carers. The support network offered advice, understanding, empathy and reassurance among carers. Family carers highlighted the Café as being the first communal space where they could sit down together and discuss dementia. The Café provided an open, honest and neutral venue where family members could openly discuss caring issues with people who had been through similar experiences:

*When you're battling with the same things, issues of personal care or whatever the things that are tricky and people would advise... I didn’t experience any support anywhere, apart from Alzheimer’s Café, in just talking about it.* **-** Carer 2
*I’ve been through it, it’s very, very difficult for carers, it really is and, you know, you try so hard to be patient and do all the things you’re supposed to do but it’s not easy, it’s not easy. And it’s only when you go through it that you know how difficult it is. And the other people there [at the Alzheimer Café], the other carers will tell you, will say the same thing.* **-** Carer 5
*Very hard to open sometimes at any meeting, I think. And then once you start, somebody else asks, and the more you get more comfortable, but sometimes you’d be dying to ask something but you’re afraid, you know? But then you think why should you be afraid? We're all in the same boat. You know, that's why we're all here you know to help each other. Or to find a bit of comfort you know. -* Carer 7

Through shared experiences of dementia, attendees formed connections with one another and developed friendships. These friendships often extend beyond the Alzheimer Café, indirectly creating support networks for people with dementia and family carers:

*I can think of two ladies particularly that I still keep up with because we just kind of got to know each other and share and swap phone numbers and I learnt from them, they would tell me how they were getting on or what they’d found you know how to approach all sorts of things, that was a real benefit, huge, huge.* **-** Carer 4
*What gets you through this is friends, and we made an awful lot of friends in the Alzheimer Café.* **-** Carer 6

It was important for carers that community extended to the person with dementia as well as themselves. This inclusion enables an increased understanding of dementia to take root within the broader caring community. This was evident in the data, indicating an acceptance that everyone was there because of the person with dementia, this shared experience helped to bond people together:

*People got agitated, you know people with dementia. It was just accepted because you knew it was part of the dementia you know, no more than Timothy ^1^ once he started. And I don’t think anyone was as bad as Timothy behaviour wise. -* Carer 7
*That was the focus of it, was the person [with dementia] in the middle of this, and then because of that person, a circle support group was going to be around that. That’s what I kind of felt* **-** Carer 2

Family carers also highlighted the potential the Alzheimer Café offered for wider community engagement as well as acting as a support for families and people with dementia. Despite recommending that other community groups create links with the Alzheimer Café, fear and stigma were barriers to this wider community engagement. Advertising the Alzheimer Café as a general community support was suggested to create engagement among the wider community:

*We have been recommending it [wider community involvement], I’m a member of a retirement group, that was one of the things I recommended but nothing came from it. I don’t know. I’d say there’s kind of fear and people just don’t want to get involved. -* Carer 9
*Well I don’t know how it’s advertised; I get a text you know. Maybe if it’s in the local paper or something that there’s a meeting…… But I think it shouldn’t just be just for dementia families, I think if you could get people from the community to go to it as well, they would learn a lot. And respect people that have dementia as well. Open it up yeah. -* Carer 7

Family carers also felt responsibility towards the Alzheimer Café. In one site, family carers highlighted that they continued attending to support the Café and keep it running, as the Café was an important resource for family carers and people with dementia. Family carers supported the Café through continued attendance and other contributions, such as collaborating with guest speakers to give talks or presenting on the Alzheimer Café externally:

*Yeah, I like to like to support it as well. Yeah, you know, and I felt like you can't just stop because Timothy went into a nursing home you know that wouldn’t be fair. -* Carer 7
*She’s giving the next talk and were going to be the guinea pigs in it because were using the technology, we’ll have a bit of fun with it. -* Carer 9
*I keep coming because if these things aren't supported the next thing is they dwindle away and that’s it. People are wondering where are they gone and what happened? Maybe they got sick of running them and think that no one is making use of this service at all. If that happens all is gone. -* Carer 8

Interestingly, this sense of ownership and responsibility towards the Alzheimer Café was particularly prevalent in a site where the Café had temporarily closed for a year due to a change of roles for the Café co-ordinator:

*I love to support it while it’s in it. Like, it was off for a year here with changes out in the main office. I used to ring them on and off and see if it would start again. Thank god they started it up again, I hope it doesn’t go off again for the good of the people. -* Carer 8

### Atmosphere

Cultivating a warm and welcoming atmosphere is instrumental in the overall success of an Alzheimer Café. Family carers strongly articulated that the Alzheimer Café was one of the first opportunities they had to discuss and explore dementia in a ‘normal’ environment.

Prior to the Alzheimer Café, some family carers felt their experiences of discussing dementia had been cold, unsympathetic and clinical. In comparison, the Alzheimer Café fosters a ‘warm welcoming’ environment where concerns were met with empathy and understanding by staff, volunteers and peers:

*Nobody wants formal especially in a thing that everyone’s going through so much in their head. You know, they don’t need to be pressurised. -* Carer 7
*It didn’t seem like a clinic, it didn’t seem like anything kind of scary for her, it was just a chat and everybody was on first name terms and that all really helped as well, we were all the same and it was just sharing tea and chat.* **-**Carer 4
*It was such a change to go to the Café after having to deal with the consultants and GP who thought John was too young to have dementia and actually have somebody listen and just be so warm and normal.* **-** Carer 6

Family carers reported an atmosphere of equality within the Café where people with dementia were treated as equals and their personhood supported. This was contrasted with other environments where people with dementia and family carers often felt stigmatised or disparaged:

*They know that even no matter what state their loved one is going through they’ll be acknowledged, helped, supported, minded and that’s great because that doesn’t happen in very many places.* - Carer 4
*We don’t make a fuss, we just get on with life, just every day normal life. When we get there everyone is normal, and everyone is fine and it’s just nice to treat people as normal.* **-** Carer 3
*Friendly and very welcoming. And for the likes of Timothy [has dementia] they were just lovely people and treated him normal you know. -* Carer 7

In terms of logistics that impacted the Café atmosphere, environmental design aspects were highlighted as important in developing atmosphere; notably the size of the room, chairs, table design, tablecloths and having teacups. Location centrality and accessibility were also important considerations. It was also highlighted that transportation was a major issue for some people who may be interested in attending:

*Make it a bit more… that everyone is kind of together, the coffee machine is together and everyone is chatting together.* **-** Carer 3
*I would be the first person to arrive at the café and it was so lovely with the nice china and lovely people.* **-** Carer 6
*The other bus provider would bring us to our door but when they lost the contract, the new crowd changed the rules. There’s plenty of people not on our route and they were coming here to things and they can’t anymore. You have to live either in a big village or a town, if you’re in between how’re you going to do that. -* Carer 9

### Activities

Family carers discussed the challenges of engaging in meaningful social activities. In some cases, family carers and people with dementia had become disengaged from activities they previously found enjoyable. A mixture of fear and uncertainty from the person with dementia, a lack of options for socialising and a lack of understanding in the general community reduced socialising opportunities for family carers and people with dementia to enjoy together. The Alzheimer Café model provided an activity and outing for carer and people with dementia to engage in together:

*There’s very little for Alzheimer’s people to do, we’re very limited.* **-** Carer 3
*‘Come on and we’ll do something’, she’d say, ‘No I don’t want to’, and I know now, a lot of it was fear, going to things and she was withdrawing even from her own brothers, things like that because I knew – I know now that she was afraid of going places where she’d get too many questions about things that she knew she couldn’t really answer.* **-** Carer 4
*There was a while there where Jenny [has dementia] never left the house for about 6 weeks or 2 months. The next thing then she got encouragement to go out and now we go to all these things. Now we leave the house once a day. At least. And we wouldn’t have looked forward to this once a month only it was helpful and useful, that’s the way I see it anyway. -* Carer 9

The opportunity to engage in activities together outside of the home was important in maintaining the relationship between the family carer and person with dementia:

*It was good that the two of us could go to that and it was just something to do really for a couple of hours because he didn’t do anything much really. Well he used to go for walks and that but towards the end he wouldn’t and that’s the only thing we kind of did so it was very good.* **-** Carer 5
*It was a safe place to bring people who had it [dementia] as well. And I kind of felt with some families, they said, ‘This is our outing, this is where we can go together in a different venue and just come out’. The only thing I always… I’m always sorry in my own head that I didn’t… I hadn’t started earlier that I could have brought Mam.* **-** Carer 2

One family carer spoke extensively about how the Alzheimer Café had been a support to him following the death of his mother who had had dementia. This carer had not previously attended the Café and was experiencing high levels of social isolation and uncertainty following his mother’s passing:

*I got it very hard to go out places afterwards. At the time I do now. Because you have no one to go with, you’re on your own. -* Carer 8
*They welcomed me with open arms and attended to me. I reckon I had a new lease of life after the death, I had someplace to come. Someplace to look forward to. -* Carer 8
*And that was another great help to me because I had something to look forward to that was in four weeks’ time say that I could go to and meet people out like. -* Carer 8

### Information

Several different information outlets were highlighted as important for family carers. A variety of channels were used to disseminate information, primarily through guest speakers, other attendees, healthcare professionals etc. The variety of inputs provided a knowledge platform encompassing a wide range of topics associated with dementia.

Guest speakers delivered informative talks on different topic areas. These talks were largely well-received and of high quality. Family carers found speakers to be a particularly useful mechanism to develop awareness of the broader landscape of dementia services (legal areas, health services etc.). Psychosocial aspects, such as emotional well-being and self-care, were also of interest to carers:

*All kinds of information, what was available, what help was available, I didn’t know anything about home help or Carer’s Allowance, any of those things, I didn’t know anything about it. So that was great to know about all these things and there was always some different speaker coming to talk about them.* **-** Carer 5
*I liked the Café because it’s a steady ship, I feel like I’m learning real things. And then having the speaker as well to introduce topics to talk about. People ask questions and I listen, I find that very good.* **-** Carer 1
*I think that it was outstanding to set it up, outstanding just for the information alone. Like you could hear Dr. …… or anybody come and give a talk, she’s a doctor in the hospital. And she’s an expert in dementia and you can hear other people talking like chemists and giving advice for people in it. Everything fits into place and people are warned of the pitfalls coming in before them. -* Carer 8

Peer information exchange consisted mainly of informal conversations among family carers. These conversations often developed narrative reconstructions of events or learning experiences, offering guidance when dealing with sensitive situations or giving practical advice to families when navigating and accessing health and social care supports.

*Got to know about maybe just even practical things about how other people [family carers] had access to things through their own public health nurses and things like that, what approach they took, bearing in mind that we were all living in different areas and each of the health service boards work slightly differently.* **-** Carer 4
*I think it's good to be there. And then somebody they'll say something, and you’ll think god that's me. You know, you can think about things differently you know what I mean? And you think, oh, maybe I should have done that, or maybe shouldn't, you know, but it's only when you hear people open up to chat. -* Carer 7

The Café often provided an opportunity for people with dementia and carers to meet with health and social care professionals. Being able to discuss a sensitive matter with a healthcare professional and having the opportunity to do so in private was important for attendees. Empathy and understanding were very important in these discussions:

*I could talk to people they have a stand with information leaflets and people coming in talking- nurses, OTs [occupational therapists], doctors, consultants, solicitors etc. After the first Café I spent the 4 weeks waiting to go back, I couldn't wait to find out more.* **-** Carer 6
*Families would have taken over the consultant or the social worker or the community nurse, and left the person they had come with, for them to have a private conversation around things, situations that were delicate.* **-** Carer 2
*Yeah, there are very good, very, very professional. You know. Well, I didn't ask much up the meetings, but I was able to go and have a private meeting with her and Timothy, you know, if you need to know anything, you know. -* Carer 7

Educating and raising awareness of dementia among less involved family members was a challenge for some carers. Family carers would often encourage other family members to attend the Alzheimer Café to learn about dementia from an unbiased source:

*My brother did come a couple of times with me, he valued it and he felt it was well run, I think again he acknowledged then that you know mum was where she was and was beginning to see that we were going to have to address certain issues but before he didn’t want to address, there was no point in me saying it, he needed to hear it from somebody else, so that was definitely helpful.* **-** Carer 4

## Discussion

Community, atmosphere, information and activity were identified as core features of the Alzheimer Café for family carers. On their own and in combination, these features have the potential to produce wider impacts in family carers’ lives. These features can help to build family carers’ capacity to manage an array of new social, environmental and cultural challenges associated with dementia. While it is important that the Alzheimer Café is enjoyable, has useful information and is supportive, it is equally important these features generate sustained improvements for family carers external to the Alzheimer Café, as is suggested by our analysis. This moves away from the prevalent understanding of ‘psychosocial’ as an intervention encompassing psychological or social components with benefits for immediate well-being, to viewing psychosocial as a very broad concept encompassing the wider context and implications of caring for a person with dementia ( Innes, 2009). As well as addressing subjective needs, psychosocial interventions can create wider social change for family carers. For example, Alzheimer Cafés identify wider systemic challenges experienced by family carers (health, social care, economic and/or legal matters) and give individuals tools that enable them to better navigate these systems. Family carers also highlighted that the Alzheimer Café could potentially influence stigma and understanding of dementia in the wider community through public engagement avenues. These effects are often lost in the evaluation of other psychosocial programmes given the tendency to focus on immediate impacts which are quantitatively measurable.

Similar to other research ( Akhtar *et al*., 2017; Dow *et al*., 2011; Greenwood *et al*., 2017), family carers described the Alzheimer Café as a warm, welcoming environment where they could access information, enjoy various activities and build support networks with other family carers. In contrast with limited timeframe psychosocial programmes, the Alzheimer Café has the potential to act as a long-term sustainable information and communication hub. Family carers also discussed the Alzheimer Café as a potential vehicle for wider community engagement. To the knowledge of the authors, no research has investigated relationships or perceptions between the wider community and Alzheimer Cafés. Other initiatives such as Science Cafés have worked with members of the general public to identify scientific topics of interest within the wider community ( Ahmed *et al*., 2014). Using informal Café methodologies, Science Cafés have successfully increased awareness and understanding of obesity, dementia, breast cancer and technology. Based on the current research and pre-existing research, there is potential for integrating public engagement elements into the Irish Alzheimer Café to reduce community stigma experienced by family carers and people with dementia. However focused research would be necessary to ensure that public engagement would not undermine the pre-existing support aims for people with dementia and family carers.

Family carers discussed several information avenues within the Alzheimer Café including guest speakers, other attendees and healthcare professionals. The opportunity to learn about different aspects of dementia from several sources and apply this information to navigate the broader service systems was important. Information from guest speakers was especially useful in relation to healthcare, finances, legal matters and self-care, serving to make family carers more confident in navigating the economic and social aspects of their role. Information exchange between family carers centred around narrative reconstructions of events or learning experiences, facilitating detailed knowledge exchange and reflective practices. Informal conversations better prepare family carers for meetings with formal practitioners by adjusting their expectations, comparing experiences, and/or contextualising their situation ( Carpentier & Grenier, 2012).

The benefits of the social network of family carers within the Alzheimer Café was not confined to information exchange purposes. Emotional support was an important feature of the Alzheimer Café experience. Many people report reduced social participation due to their role as a family carer ( Nay *et al*., 2015). Prioritising the needs of the person with dementia, reduced capacity for spontaneity and stigma are often key factors underlying this loss of social participation. In response, the Alzheimer Café facilitates a community compatible with caring lifestyles, offering reassurance and alleviating carers’ isolation. Strong emotional support from professionals and peers can allow family carers to continue in their role with greater confidence ( Brooker *et al*., 2017; Gaugler *et al*., 2009). Despite similar objectives, other interventions such as befriending, have had limited success in building social networks among family carers ( Charlesworth *et al*., 2008). This could be attributable to time constraints imposed on targeted interventions, developing friendships is difficult within a set time period ( Charlesworth *et al*., 2008). In contrast, the Alzheimer Café occurs regularly over a long-term period facilitating meaningful connections organically over time. Our research found these friendships sometimes extend past the monthly meetings and become embedded in family carers’ overall social support system.

Alongside information and community, atmosphere was a core aspect of the Alzheimer Café. Prior to attending the Alzheimer Café, family carers had mainly engaged with dementia in clinical settings. Unfortunately, many of these interactions were characterised by a lack of empathy and understanding which contributed to carers’ feelings of isolation. In contrast, the Alzheimer Café provided an understanding, empathetic and safe atmosphere where family carers could explore and develop supportive and empowering emotional responses to dementia. Another study highlighted similar benefits, reporting the Alzheimer Café provided a setting where carers could normalise, adapt to and learn about dementia ( Greenwood *et al*., 2017). This is an important consideration as processing a diagnosis contributes to developing family carers’ resilience and well-being ( Duggleby *et al*., 2009; Donovan & Corcoran, 2010; Shim *et al*., 2013).

The Alzheimer Café provides a space where family carers and people with dementia to enjoy meaningful activities together. For a variety of reasons, people with dementia and family carers often report they no longer engage with previous hobbies or interests. According to our interviewees, the Alzheimer Café provided opportunities for social interaction and relationship building with the person they care for and other family members, enjoying activities together outside of the home has been shown to foster social bonds within families and within their wider social networks. Previous research proposed social activities, such as ‘eating out’ ( Cassolato *et al*., 2010), are important in facilitating interconnectedness, meaningful communication, greater feelings of intimacy and positive affect among friends and family networks.

How different is the Irish Café to the experience in other countries? The American Alzheimer Café model is solely framed as a social outing in a community-based location where dementia ‘is not in the room’. Although the Irish Alzheimer Cafés in this study were based in community settings and incorporated designated time for socialising at tables, unlike the American model they also had a strong informational and educational focus. Family carers in this study identified information and education as core aspects of the Alzheimer Café alongside activities and atmosphere. Similar to the European model, open discussion of dementia was encouraged and each site in this study had regular guest speakers on different topics related to dementia. Health and social care professionals were present at each of the three Irish Cafés to provide information of advice or signposting to people with dementia and family carers. The Irish Cafés appear, therefore, to follow a similar structure to the European model- designated social time, guest speaker, and the presence of health and social care professionals. However, the European model advocates adherence to 33 quality control criteria (QCC), which is not part of the Irish approach.

Consequently, there may be a lot more heterogeneity in these Irish Alzheimer Cafés than you find in the generic European model – certainly Irish Cafés in this study did not always rigidly follow the same format. One of the three sites had music and dancing following the guest speaker, another site sometimes replaced the guest speaker with a festive social event (e.g. summer or winter celebrations), another Café would sometimes have speakers on non-dementia related topics. There was strong evidence of variability and responsiveness to the needs of those attending. This flexibility or non-adherence to the European QCC among the sites in this study was a distinguishing feature from the European model. In this regard, despite not having a dedicated counsellor, Irish Alzheimer Cafés in this study most closely resemble the Australian Memory Lane Café model used in Victoria, rather than the European or American model. The Australian Memory Lane Café model has an education focus, unlike the American Café, but does not strictly adhere to the QCC set out by the European Alzheimer Café model, sometimes even interchanging guest speakers with musical entertainers ( Dow *et al*., 2011).

## Conclusion

Our research found the Alzheimer Café has direct and indirect effects on the well-being of family carers. As a single-level intervention, the Alzheimer Café facilitates emotional exploration, development and normalisation. It offers information on maintaining well-being and can improve family carers’ understanding of dementia. Through an ecological systems lens, these single-level effects translate into multi-level impact on family carers’ lives, including relationship building within families, engaging with community supports and developing new social networks. The Alzheimer Café identifies challenges faced by family carers and integrates this understanding into their social programme. On the wider systems level, the Alzheimer Café offers family carers the opportunity to acquire skills through information sharing avenues which could lead to improved navigation of health, social care, and legal systems.

As is evident above, a framework facilitating exploration of psychosocial outcomes beyond immediate consumption benefits is an important tool when evaluating psychosocial interventions. This paper gives an insight into what a psychosocial support can be and the wider benefits it can bring to peoples’ lives. Given the rising popularity of psychosocial supports as an avenue to support family carers, it is important to give examples of how psychosocial supports can be mobilised to have wider impact. We recommend that future evaluations of Alzheimer Cafés and other psychosocial programmes could be enhanced through use of ecological systems frameworks.

### Limitations

This study was conducted with a convenience sample of three Alzheimer Café sites in Ireland and therefore may not give a complete picture of activities in all the estimated 20 Alzheimer Cafés in Ireland. This was a small scale exploratory study which aimed to gain a deeper understanding of Irish family carer perspectives of the Alzheimer Café- given the aims of the study the authors feel the convenience sampling framework was appropriate. Consequently, however, this paper should serve as a foundation for further research on family carers perspectives of the Alzheimer Café, rather than be seen as representative of all family carers attending Alzheimer Cafés in Ireland. In fact, the research team has used the findings of the current paper to inform the development of a national survey of all Alzheimer Cafés in Ireland. Therefore, following the publication of the national survey, it may be possible to identify a wider range of Alzheimer Café models in operation across Ireland. If that is the case, the findings of the national survey may usefully inform the development of a more in-depth comparative qualitative study exploring family carers perceptions of different Alzheimer Café models in Ireland. Another limitation of this study is the small number of participants interviewed. However, three participants in each site accounted for 20% of the total family carers attending an average café on any given day.

## Data availability

The data in this study, consisting of interview transcripts and field notes, cannot be de-identified sufficiently to ensure the anonymity of the research participants. Consequently, this data cannot be made available publicly. However, if the research team is assured interviewees' anonymity can be protected, data from the current research is available for further research upon reasonable request.

To access the data, please contact the corresponding author ( a.teahan1@nuigalway.ie) or the Principal Investigator ( eamon.oshea@nuigalway.ie). Researchers will be asked to provide a short proposal on how the data will be used before access is granted.

## Notes

^1^Pseudonyms have been used to protect the identity of the interviewees.
