# Supplementary material for: Family carers’ perspectives of the Alzheimer Café in Ireland
Source: HRB Open Res. 2020 Aug 17;3:18. Originally published 2020 Apr 30. [Version 2] doi: 10.12688/hrbopenres.13040.2 (PMC7243203; doi:10.12688/hrbopenres.13040.2)
Supplement: Peer review history

# Peer review: Reviewer response for version 2

**DOI:** 10.21956/hrbopenres.14242.r27828

**Published:** 2020-08-24

**Authors:** Briony Dow

**Affiliations:** 1 National Ageing Research Institute, Melbourne, Australia

I am happy with the revisions made and the explanation regarding the role of Bronfennenner's model in the study.

Is the work clearly and accurately presented and does it cite the current literature?

Yes

If applicable, is the statistical analysis and its interpretation appropriate?

Not applicable

Are all the source data underlying the results available to ensure full reproducibility?

No source data required

Is the study design appropriate and is the work technically sound?

Partly

Are the conclusions drawn adequately supported by the results?

Partly

Are sufficient details of methods and analysis provided to allow replication by others?

Yes

Reviewer Expertise:

Social gerontology.

I confirm that I have read this submission and believe that I have an appropriate level of expertise to confirm that it is of an acceptable scientific standard.

# Peer review: Reviewer response for version 2

**DOI:** 10.21956/hrbopenres.14242.r27830

**Published:** 2020-08-18

**Authors:** Louise Hopper

**Affiliations:** 1 School of Psychology, Dublin City University, Dublin, Ireland

The updates the authors have made to this manuscript have successfully addressed the gaps highlighted in previous reviews. The addition of Table 2, which documents frequency of attendance at the Cafe is particularly helpful, as it the discussion of the different Alzheimer Cafe models. A better understanding of how the Irish approach fits into the international context is achieved.

 Overall, this is a very interesting and well written paper and it will inform further study in the area.

Is the work clearly and accurately presented and does it cite the current literature?

Yes

If applicable, is the statistical analysis and its interpretation appropriate?

Not applicable

Are all the source data underlying the results available to ensure full reproducibility?

Partly

Is the study design appropriate and is the work technically sound?

Yes

Are the conclusions drawn adequately supported by the results?

Yes

Are sufficient details of methods and analysis provided to allow replication by others?

Yes

Reviewer Expertise:

I conduct research in the dementia field, including research with people with dementia, their family carers and with dementia service providers. I am also co-founder and Co-chair of the Alzheimer Cafe Leixlip.

I confirm that I have read this submission and believe that I have an appropriate level of expertise to confirm that it is of an acceptable scientific standard.

# Peer review: Reviewer response for version 1

**DOI:** 10.21956/hrbopenres.14136.r27372

**Published:** 2020-05-27

**Authors:** Briony Dow

**Affiliations:** 1 National Ageing Research Institute, Melbourne, Australia

I really enjoyed reading this paper. It is a well-written description of a qualitative study of family carers’s experiences of Alzheimer’s cafés. I think it is an important topic as there is still a great need for programs and services that support family carers in the very demanding and often isolating role of caring for a person living with dementia.

 I have very few editorial comments – there are only one or two typos in the manuscript. However, I was not convinced about the ecological systems approach. While carers were asked about the impact that the cafés had on their social lives and use of services, this is still a very individualistic approach, involving only individual and microsystem levels of ecological systems theory. If the authors wish to use this framework, it should be properly described, including how they have adapted Brofenbenner’s framework and used it to guide the design of the study and interpret the findings. How are each of the five level systems reflected in the interviews with carers? How do the broader system elements come into play?

 I was also looking for a stronger rational as to why it is important to know what the carers’ experiences of Alzheimer’s cafés are. While this might be self-evident to the authors, I think this should be drawn out more, including the broader public policy issues involved.

Is the work clearly and accurately presented and does it cite the current literature?

Yes

If applicable, is the statistical analysis and its interpretation appropriate?

Not applicable

Are all the source data underlying the results available to ensure full reproducibility?

No source data required

Is the study design appropriate and is the work technically sound?

Partly

Are the conclusions drawn adequately supported by the results?

Partly

Are sufficient details of methods and analysis provided to allow replication by others?

Yes

Reviewer Expertise:

Social gerontology.

I confirm that I have read this submission and believe that I have an appropriate level of expertise to confirm that it is of an acceptable scientific standard, however I have significant reservations, as outlined above.

# Response

**Published:** 2020-08-07

**Authors:** Áine Teahan

**Affiliations:** Centre for Economic and Social Research on Dementia, NUI Galway, Ireland

Thank you for reviewing this paper and for your insightful comments. We have integrated your suggestions into the paper in the introduction and methods sections.

Explicitly integrating Bronfenbrenner’s five levels into the interview guide was considered in the early design stages of this study. However, following consultations with family carers and other researchers, it was decided that asking family carers direct questions on the relationship between the Alzheimer Café and the five ecological systems levels was too abstract particularly for exosystem and macrosystem levels.

As a result, a more organic approach was taken with the interview guide where, using prompts, family carers identified wider social issues (system organisation, culture, society, public policy etc.) they had experienced while caring for a person with dementia. Through reflection of their own experiences family carers considered if the Alzheimer Café had, did not have, or could have an impact in these contexts. For example, although not directly asking about the healthcare system organisation and policies, asking family carers how they engaged with healthcare professionals opened up discussions about the broader health system, specifically how family carers fared navigating this system, if the Alzheimer Café had any impact etc. As you have highlighted, through the question on social life, we also attempted to draw some response from respondents on community and societal stigma.

The aim of this research is to explore the experiences of family carers of people with dementia attending Alzheimer Cafés in Ireland. Bronfenbrenner’s framework provided very useful conceptual guidance, particularly in relation to exploring how immediate benefits of an Alzheimer Café could translate into broader impact for family carers. However, mapping the Alzheimer Café within this framework proved to be beyond the scope of the work. We acknowledge that we could have done more to draw out specific ecological systems implications, but teasing out the broader implications would require a separate paper. Consequently, we have rewritten part of the methods esction to highlight that while this research was certainly influenced by ecological systems theories, the main aim was to identify family carers own perspectives of how the Alzheimer Café created impact (if it created impact) in their lives.

# Peer review: Reviewer response for version 1

**DOI:** 10.21956/hrbopenres.14136.r27369

**Published:** 2020-05-21

**Authors:** Hajime Takechi

**Affiliations:** 1 Department of Geriatrics and Cognitive Disorders, Fujita Health University School of Medicine, Toyoake, Japan

This paper provides a qualitative analysis of the significance of dementia cafes in Ireland, primarily from a family support standpoint. Nine family caregivers with experience of attending three dementia cafés were interviewed and analyzed. Overall, this paper is well written. I would like to raise 2 points for the clarification of this paper.

 Some new findings in this paper were mentioned in the discussion, but it would be better if they were also mentioned more clearly in the abstract, as it is difficult to understand what is meant by the four core features, especially activity.

 Although Alzheimer Café (AC) in Ireland is described, it would be preferable if the status of dementia care other than AC and community resources in Ireland and their positioning in the context of the national strategy had been mentioned. It has also been noted that the AC model varies by region, but it would be desirable to have some consideration of whether this is influenced by existing local resources for dementia care or whether it is related to other factors.

Is the work clearly and accurately presented and does it cite the current literature?

Yes

If applicable, is the statistical analysis and its interpretation appropriate?

Not applicable

Are all the source data underlying the results available to ensure full reproducibility?

Partly

Is the study design appropriate and is the work technically sound?

Yes

Are the conclusions drawn adequately supported by the results?

Partly

Are sufficient details of methods and analysis provided to allow replication by others?

Yes

Reviewer Expertise:

Geriatrics, dementia research.

I confirm that I have read this submission and believe that I have an appropriate level of expertise to confirm that it is of an acceptable scientific standard.

# Response

**Published:** 2020-08-07

**Authors:** Áine Teahan

**Affiliations:** Centre for Economic and Social Research on Dementia, NUI Galway, Ireland

Thank you for your useful comments and international perspective. We have addressed your suggestions in the abstract and introduction sections.

# Peer review: Reviewer response for version 1

**DOI:** 10.21956/hrbopenres.14136.r27371

**Published:** 2020-05-21

**Authors:** Anthea Innes

**Affiliations:** 1 Salford Institute for Dementia, University of Salford, Manchester, UK

This is a timely and well written article exploring the perceived benefits of attending Alzheimer Cafes (3) in Ireland. It is well contextualised in the literature and the approach taken to data collection is clearly articulated. A fuller account of the process of the development of the themes would be helpful should others wish to conduct similar research looking at variations of the dementia café movement in different countries (or even the same country).

 I am curious to know how much divergence there was in the cafes included in the study and also how similar or different each was to the 'European' model in particular given Ireland's EU status.

 The paper provides a useful framework to consider the benefits of participating in such a group. There is however some missing detail as it appears that research participants, the cafe members, may not have attended often - this detail is missing, simply states that a minimum of 1 café had been attended in the last month.

 The paper will be of interest to other psychosocial researchers seeking to explore the benefits and impacts of attending similar groups, and also to those tasked with delivering such groups in practice.

Is the work clearly and accurately presented and does it cite the current literature?

Yes

If applicable, is the statistical analysis and its interpretation appropriate?

Not applicable

Are all the source data underlying the results available to ensure full reproducibility?

Partly

Is the study design appropriate and is the work technically sound?

Yes

Are the conclusions drawn adequately supported by the results?

Yes

Are sufficient details of methods and analysis provided to allow replication by others?

Partly

Reviewer Expertise:

I have conducted research in the dementia field for the last 25 years. I am particularly interested in psychosocial research, and the views/experiences of people living with dementia/care partners.

I confirm that I have read this submission and believe that I have an appropriate level of expertise to confirm that it is of an acceptable scientific standard.

# Response

**Published:** 2020-08-07

**Authors:** Áine Teahan

**Affiliations:** Centre for Economic and Social Research on Dementia, NUI Galway, Ireland

Thank you for your valuable comments and for reviewing our paper. We have provided additional details in the methods, results and discussion sections of the updated paper to address your comments.

# Peer review: Reviewer response for version 1

**DOI:** 10.21956/hrbopenres.14136.r27370

**Published:** 2020-05-18

**Authors:** Louise Hopper

**Affiliations:** 1 School of Psychology, Dublin City University, Dublin, Ireland

In this manuscript, the authors take an ecological systems approach to examine family carers’ perspectives of the Alzheimer Café in Ireland and an evaluation of how benefits deriving from café attendance may translate to daily life. The variety of Alzheimer Café models in Ireland are also discussed.

 The manuscript has several strengths including the novel ecological-systems approach, the overview of different international Alzheimer Café models, clearly presented results and insightful discussion, and the general presentation and clarity of written expression.

 However, some clarifications are required and some limitations influence the transferability of these findings:

1. As the authors note, the ad-hoc development of Alzheimer Cafes in Ireland has led to a situation where there is considerable diversity across the 20 existing cafes. A variety of other ‘Alzheimer Type’ cafes also exist in Ireland and the lines are blurred as to the differences between the two – sometimes these differences are structural in nature – sometimes they relate to the content and guiding approach to the Café – but at other times it is merely a desire to move away from the term Alzheimer’s. It would be important to clarify that this is the context within which the national Alzheimer Café Coordinator is trying to work as it adds considerable complexity to the desire to create a governance model that works for all cafes.
2. The stated aim is to examine how the benefits of café attendance translate into broader life, yet the inclusion criteria for family carers is that they need to have attended just one café in the 6 months prior to interview. There is no discussion of the overall frequency or duration of attendance, either as an influence in the recruitment process or when participant characteristics are presented in the results. I would question the ability of someone to speak to the broader impact of just one instance of a behaviour. It would be helpful if the authors could clarify the breadth of experience their participants had.
3. The three cafes were chosen for convenience, yet as the author’s state, there are many different models in use across Alzheimer Cafes in Ireland. Could the authors please clarify (a) how many different models were covered in the three cafes selected and (b) why purposive sampling of Cafes was not considered? It is difficult to determine the breadth of approaches experienced by the family carers without this information.
4. In the Discussion, it would be helpful to elaborate on the main ways in which Irish cafes (a) resemble the Australian model and (b) differ from the European and American models, and to evaluate what the likely implications are as a result.
5. The idea of public engagement is an interesting one. In addition to potentially reducing the stigma that is still often associated with dementia, it also provides a mechanism for building awareness of what the Alzheimer Café has to offer. It is certainly prudent to ensure that supporting people with dementia and their family caregivers is not undermined, but I would question the use of the phrase ‘significant research’. Perhaps ‘focused research’ would be a better alternative?
6. The authors clearly acknowledge many of these limitations in the Discussion. The national survey of Alzheimer Cafes in Ireland will go some way towards addressing these limitations, but it will lack the depth that qualitative research can bring. Perhaps the results of this research could argue more strongly for the adoption of a mixed-methods approach where a subset of participants with a wider range and duration of café experience are drawn from cafes representing the range of models in operation, so that we can learn more from their experiences and positively influence the models used by new and existing cafes.

This informative study takes a novel and welcomed approach to examining the impact of a heterogeneous community-based psychosocial interventions. The findings will not only broaden our understanding of the benefits of Alzheimer Cafes for family carers, but also demonstrate the value of looking beyond immediate impacts when evaluating psychosocial interventions.

Is the work clearly and accurately presented and does it cite the current literature?

Yes

If applicable, is the statistical analysis and its interpretation appropriate?

Not applicable

Are all the source data underlying the results available to ensure full reproducibility?

Partly

Is the study design appropriate and is the work technically sound?

Yes

Are the conclusions drawn adequately supported by the results?

Yes

Are sufficient details of methods and analysis provided to allow replication by others?

Yes

Reviewer Expertise:

I conduct research in the dementia field, including research with people with dementia, their family carers and with dementia service providers. I am also co-founder and Co-chair of the Alzheimer Cafe Leixlip.

I confirm that I have read this submission and believe that I have an appropriate level of expertise to confirm that it is of an acceptable scientific standard, however I have significant reservations, as outlined above.

# Response

**Published:** 2020-08-07

**Authors:** Áine Teahan

**Affiliations:** Centre for Economic and Social Research on Dementia, NUI Galway, Ireland

Thank you for your insightful review. We have addressed the majority of your suggestions in our updated paper.

In relation to comment 4 - to protect the anonymity of the interviewees we have provided only broad comparisons between the Irish Alzheimer Cafés in this study and the other international models in the discussion section.
